# Interaction of Lipopolysaccharide-Spiked Blood with Anti-Fouling Polymyxin B-Modified Glass

**DOI:** 10.3390/ma15041551

**Published:** 2022-02-18

**Authors:** Hoi Ting Wong, Alexander Romaschin, Sara Bjelobrk, Brian De La Franier, Michael Thompson

**Affiliations:** 1 Department of Chemistry, University of Toronto, 80 St. George Street, Toronto, ON M5S 3H6, Canada; hoiting.wong@mail.utoronto.ca (H.T.W.); sara.bjelobrk@mail.utoronto.ca (S.B.); brian.delafranier@mail.utoronto.ca (B.D.L.F.); 2 Clinical Biochemistry, St. Michael’s Hospital, 30 Bond Street, Toronto, ON M5B 1W8, Canada; romaschina@gmail.com

**Keywords:** lipopolysaccharide, endotoxin, anti-fouling, polymyxin B, glass beads

## Abstract

Bacterial endotoxin, also known as lipopolysaccharide (LPS), plays a major role in the initiation of sepsis, a severe inflammatory condition. Removal of the toxin from blood is one accepted method of patient treatment. Polymyxin B (PMB)-modified columns have been employed successfully for this purpose via extra-corporeal blood-flow systems that incorporate a cartridge for toxin removal. Herein we demonstrate that PMB-modified glass beads are able to reduce the presence of LPS competitively with the equivalent fiber column used in a commercial cartridge. Analysis by gas chromatography-mass spectrometry and ELISA of released fatty acids from the toxin indicates that PMB does not physically capture or significantly remove LPS from the blood samples. In reality, interaction between the surface-bound PMB and the toxin may lead to disaggregation or monomerization of LPS aggregates. As aggregates are the bioactive form of LPS, it is possible that the monomerization of these entities may be the mechanism by which their toxicity is reduced. Moreover, this work indicates that LPS monomers are stabilized subsequent to disaggregation induced by PMB.

## 1. Introduction

Lipopolysaccharides (LPS) originating from Gram-negative bacteria, also known as endotoxins, are well known to be cause endotoxemia which can be an initiator of a life-threatening medical condition of overwhelming inflammatory response called sepsis. The severity of the bacterial infection is very likely to be associated with the high concentration of LPS circulating in the bloodstream. Millions of cases of sepsis are diagnosed worldwide each year, and this medical condition is linked to high personal and economic costs due to prolonged stays in intensive care units (ICUs) and increased use of hospital resource for treatments [1]. Given the medical consequences instigated by LPS it is not surprising that there is a vast literature dedicated to its biology, biochemistry, bioactivity, detection and treatment by removal [2,3,4,5,6,7,8,9,10,11,12,13,14].

**Figure 1:**
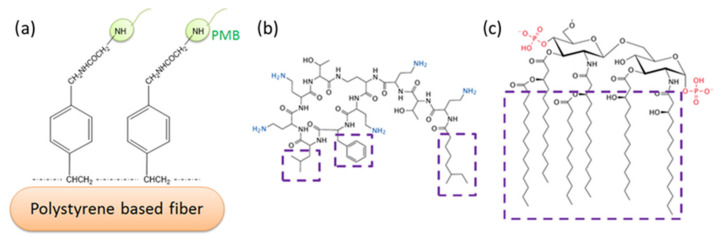
Typical PMB-immobilized polystyrene-based fiber system (**a**); proposed interaction mechanism of PMB (**b**) and Lipid A of LPS (**c**). Ionic interaction of amino groups in PMB (in blue) and phosphate groups in Lipid A (in red); Van der Waals’ interaction between hydrophobic regions of PMB and Lipid A (in purple boxes).

Due to their amphipathic properties, LPS molecules tend to form multimeric clusters in aqueous solution above a critical micelle concentration (CMC) [15]. For instance, LPS extracted from *E coli.* O55: B5 has a CMC of 38 μg/mL; when extracted from *Escherichia coli* 0111: B4 it has a CMC of 22 μg/mL [16]. Even though the molecular mass of LPS is approximately 10–30 kDa, aggregates can be as large as hundreds or even thousands of kDa [17]. Even at very low concentrations below the CMC (e.g., 10 pg/mL), LPS may still be highly aggregated without any visible signs of the LPSs breaking up into individual monomers [18]. It has been recognized that LPS aggregates in various forms, rather than monomers, are responsible for the initiation of bacterial infection [19,20,21,22]. Potential patient treatment via the removal of LPS aggregates from the bloodstream has been the subject of intense research over a number of years. Many systems have been studied for their potential utility as binding agents. Fiber-bound polymyxin B has figured prominently in research over the last twenty years (Figure 1); accordingly, we have chosen to concentrate on studies of this LPS binding agent.

In one of the earliest studies, it was shown that PMB hemoperfusion-based removal of LPS strongly influences the secretion of TNF alpha and NF-kB [23]. The presence of the latter moieties was examined using ELISA. The nature of the interaction of the LPS monomer with PMB has attracted both theoretical and experimental investigation. For example, Vesentini et al. [24] used computer models to demonstrate that both hydrophobic and hydrophilic interactions are in play, whereas experiments with isothermal titration calorimetry, differential scanning calorimetry, and X-ray diffraction indicated the importance of electrostatic interactions [25]. Researchers have also conducted numerous studies on the clinical use of PMB hemoperfusion [26,27,28,29,30,31,32]. Generally speaking, the conclusion has been reached that PMB hemoperfusion improves the conditions of patients exhibiting sepsis, although there are studies that indicate that survival rates associated with PMB hemoperfusion are not necessarily enhanced [33].

Recently, researchers have employed a variety of techniques for further examining the nature of the PMB-LPS interaction, including the employment of substrates other than polystyrene-based fiber as well as the use of anti-fouling surface chemistry. Madhumanchi et al. [34] used a plethora of methods such as UV fluorescence, NMR and infrared spectroscopies, circular dichroism and dynamic light scattering to look at the binding of LPS to PMB. Atomic force microscopy experiments have been conducted where the atomic force microscope (AFM) tungsten tip was modified by PMB and LPS avidin-attached to the usual mica substrate or in *E. coli* cultures [35]. The results of this work indicated that the binding of PMB to LPS, both in isolated monolayers and in *E. coli* cell membranes, increases surface roughness and that the unbinding force between LPS and PMB is approximately 30 pN. With regard to other surfaces for binding PMB, Cao et al. [36] used cellulose microspheres successfully, the argument being that this material offers a less expensive substrate than the conventional fiber approach. A similar argument was made with respect to studies of gellan-polylysine fibers [37]. Vorobii et al. [38] used N-2-hydroxypropyl methacrylamide (HPMA) and carboxybetainemethacrylamide (CBMAA) to produce a polymer, poly(HPMA-co-CBMAA), for PMB attachment to generate an anti-fouling surface.

**Figure 2:**
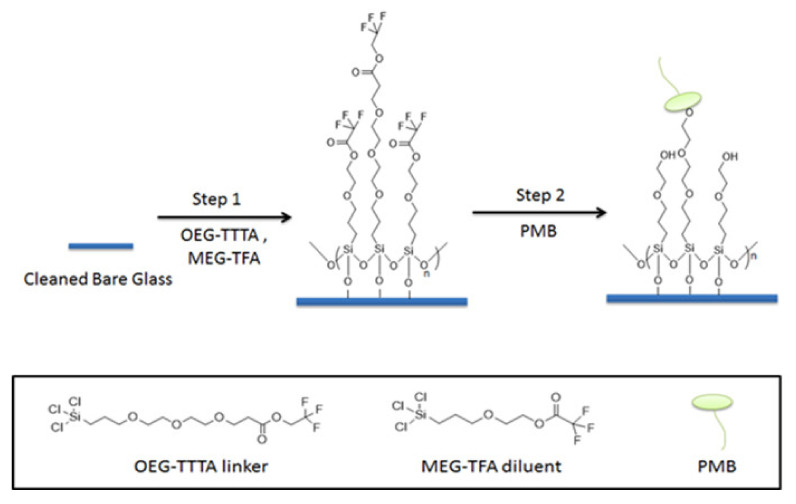
Preparation and immobilization of PMB on bare glass beads.

In the present work, we introduce borosilicate glass beads as a carrier for PMB as an alternative to, for example, an existing fiber-based system. The approach is intended to maximize the interaction of LPS and PMB via the enhanced exposure of the adsorbent compartment to blood, through increased surface area. Here we employ a previously-developed mixed monolayer based on silanization chemistry that mitigates non-specific adsorption in addition to the tandem capability for attachment of PMB [39,40,41]. For this purpose, 2-(3-trichlorosilylpropyloxy)-ethyl-trifluoroacetate (MEG-TFA) and oligoethylene glycol analog of 2,2,2-trifluoroethyl-13-trichlorosilyl-tridecanoate (OEG-TTTA) were chosen as the diluent and linker molecules, respectively, as shown in Figure 2. The specialized hydration properties of the oligoethylene moiety convey significant anti-fouling properties to surface-attached monolayers [42,43].

**Figure 3:**
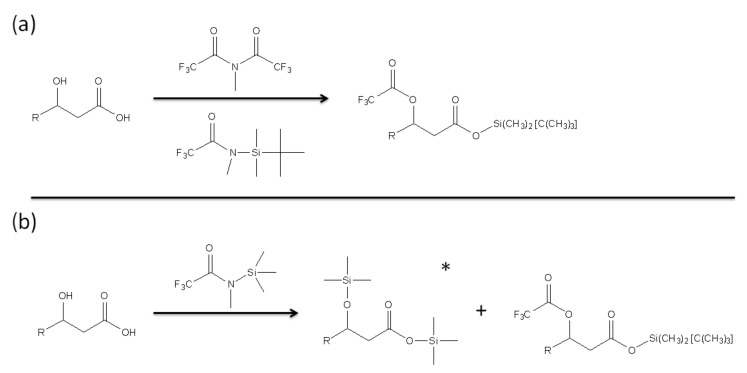
Derivatization of hydroxyl fatty acid using (**a**) methy-bis(trifluoracetamide) (MBTFA) and N-metyhl-N-(tert-butyldimethylsilyl) trifluoracetamide (MTBSTA) and (**b**) N-Methyl-N-(trimethylsilyl) trifluoroacetamide (MSTFA) as derivatizing agents. The * symbol in (**b**) indicates the major product of the derivatization (>90%).

In order to quantify the presence of free LPS and LPS that potentially binds to PMB-modified glass beads, GC-MS was employed to analyze derivatized products of 3-hydroxylauric acid and 3-hydroxymyristic acid [44]. These two fatty acids were selected as they are likely to be released by Lipid A of LPS as products of alkaline hydrolysis. The derivatizing agents were chosen for both the production of the intended separation and the ease of quantification of products (Figure 3).

## 2. Materials and Methods

### 2.1. Materials

3-Hydroxylauric acid (12:0 3-OH, ≥99%), 3-hydroxymyristic acid (14:0 3-OH, ≥98%), tridecanoic acid (13:0, ≥98%), pentadecanoic acid (15:0, ≥98%), heptadecanoic acid (17:0, ≥98%), N-Methyl-N-(trimethylsilyl) trifluoroacetamide (MSTFA, ≥98.5%), *E.coli* O55:B5 LPS (lyophilized powder collected by phenol extraction), fluorescein isothiocyanate (FITC)-labelled *E.coli* O55:B5 LPS (2–10 μg FITC per mg LPS), proteinase K (from Tritirachium album, ≥30 units/mg protein) and Dulbecco’s phosphate buffered saline (PBS, CaCl_2_, MgCl_2_-free, pH 7.4) were purchased from Sigma-Aldrich (Oakville, ON, Canada). 12 M Hydrochloric acid, 12 M sodium hydroxide, ACG grade chloroform, acetone and methanol were obtained from EMD Chemicals Inc. (Gibbstown, NJ, USA) while pyridine (extra dry with molecular sieves) was obtained from Acros Organics. Methyl-bis(trifluoroacetamide) (MBTFA) and N-methyl-N-(tert-butyldimethylsilyl) trifluoroacetamide (MTBSTA) were purchased from CovaChem (Loves Park, IL, USA). Anhydrous sodium sulfate was purchased from Thermo Fisher Scientific (Waltham, MA, USA) and ethyl acetate was purchased from VWR International (Radnor, PA, USA). 0.9% sodium chloride irrigation fluid and human albumin (USP, 25% Solution) were obtained from Baxter International Inc (Chicago, IL, USA). Human blood was collected in sterile EDTA anticoagulation vacutainers (Becton Dickinson, Franklin Lakes, NJ, USA) from healthy donors and the author at St. Michael’s Hospital (Toronto, ON, Canada) on the day of experiment and was used without delay. Toraymyxin cartridges were purchased from Spectral Medical Inc. (Toronto, ON, USA). Masterflex L/S economy pump system (Cole-Parmer, Vernon Hills, IL, USA) with easy-load II pump head along with C-Flex ULTRA L/S 24 tubing was provided by Spectral Diagnostics Inc (Toronto, ON, USA). Glassware was rinsed with chloroform and heated at 290 °C for 2 h before use to eliminate moisture and pyrogen contaminates. Sterile polypropylene tubes with screw caps or disposable culture borosilicate glass tubes (VWR International, Radnor, PA, USA) were also used to minimize the possibility of contamination from pyrogen when appropriate. Gold standard syringes (10µL FN 23/42/HP) from Agilent Technologies (Wilmington, DE, USA) were used to measure and transfer derivatizing agents which are highly sensitive to moisture.

Human blood was available from a single source through Canadian Blood Services in collaboration with St. Michaels’s Hospital, Toronto, ON, Canada. No individual donor was employed or associated with the research conducted and described in this paper.

### 2.2. Gas Chromatography-Mass Spectrometry and ELISA

For GC-MS analysis, a gas chromatograph operated with 6890N network GC system with 5975C inert XL EI/CI mass selective detector was used along with a 7683 B series auto injector for split/splitless injection (Agilent Technologies, Inc., Wilmington, DE, USA). The Agilent gas chromatograph system was equipped with a capillary column, which is made of methyl siloxane, in dimensions of 12 × 200 × 0.33 µm. The carrier gas for GC separation was helium and the linear being used was 4 mm ID tap GW along with a non-stick O-ring purchased from Agilent Technologies (Wilmington, DE, USA). 96-Well human TNFα ELISA kits (model: EH3 TNFA) purchased from Thermo Scientific (Waltham, MA, USA) were stored at −24 °C freezer and thawed at room temperature before each use.

### 2.3. Fluorescence Measurement of the LPS Binding Ability of the Fiber Cartridge

PMB-immobilized polystyrene-based fiber was withdrawn from a commercial cartridge and cut into a circle with a diameter of approximately 50 mm. The fiber cloth was then placed in a sterile petri dish with its mass determined by differential weighing. 25% Human albumin solution was diluted 10-fold with 0.9% sodium chloride solution. The resulting solution containing 2.5% albumin (5 mL) was used to dilute 200 μL of fluorescein isothiocyanate labelled LPS (FITC-LPS, 1 mg/mL) to a concentration of 40 μg/mL. This FITC-LPS solution was added to the petri dish containing the PMB-immobilized fiber. After covering the petri dish, the mixture was incubated at room temperature for 90 min.

Guanidine hydrochloride (GdmCl) solution (6 M) was prepared by dissolving a corresponding amount of GdmCl in pyrogen-free water while stirring on a stir plate. After incubation, the PMB-immobilized fiber was rinsed with 0.9% sodium chloride solution (50 × 5 mL), followed by rinsing with 2.5% albumin in 0.9% NaCl (50 × 2 mL), and finally rinsed with 0.9% sodium chloride (50 × 2 mL) under gentle shaking for 3 min each. Control PMB-immobilized fiber samples were prepared similarly, with the only exception being that FITC-LPS was not added. Before incubating the fiber sample in GdmCl (6 M, 5 mL) at 37 °C for 15 min, a small fragment of the sample (5 × 5 mm) was cut out for fluorescence measurement. The FITC-LPS solution was also collected after the incubation to measure its absorbance using an automatic plate reader with a test wavelength of 518 nm and a reference wavelength of 630 nm. Various solutions of FITC-LPS standard (0.005–1 mg/mL) were also prepared and their absorbances were measured for the construction of a calibration curve.

### 2.4. Adsorption of LPS on Proteinase K-treated Commercial Column

**Figure 4:**
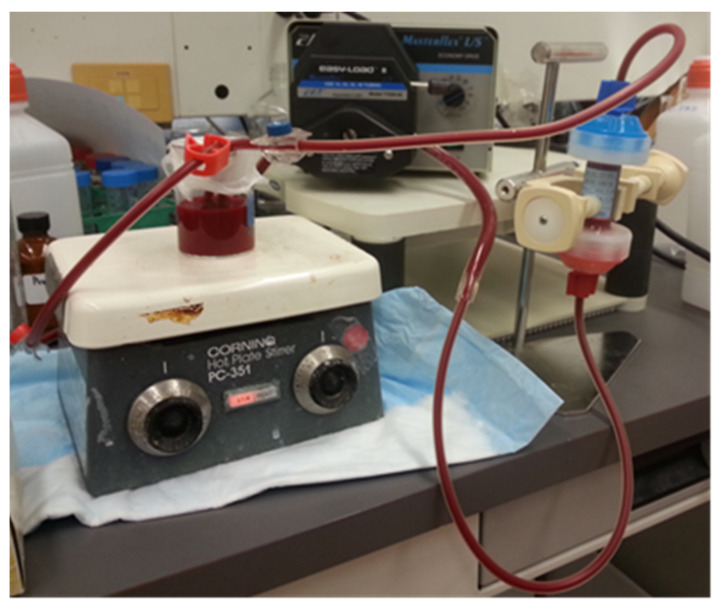
Simulation of extracorporeal LPS removal via a “baby” cartridge.

100 μg/mL Proteinase K solution was prepared by reconstituting an appropriate amount of proteinase K lyophilized powder in a 50 mL buffer containing 50 mM Tris-HCl (pH 8.0), 3 mM CaCl_2_ and 0.5% sodium dodecyl sulfate (SDS). A commercial cartridge specified for use on a baby was employed as in Figure 4.

The PMB-immobilized fiber column was first removed from the “baby” cartridge and then submerged in the prepared proteinase K buffer solution (100 μg/mL, 10 mL) at 37 °C for 12 h. The fiber column was then removed from the proteinase K solution, gently rinsed with 0.9% sodium chloride irrigation to remove any remaining solution and inserted back into the original “baby” cartridge. A Masterflex pump system was used to maintain circulation of blood at a flow rate of 80 mL/min. 80 mL of Whole blood was spiked with 170 μg FITC-LPS and was then perfused over the “baby” cartridge containing the proteinase K-treated fiber column for 90 min at room temperature. Two tubes of 5 mL blood samples were collected and centrifuged at 2500 rpm for 15 min. The plasma layers were then collected for fluorescence intensity measurement. A linear calibration curve was prepared by measuring the fluorescence intensity of a series of FITC-LPS standards covering the experimental range. All measurements were completed on an automatic plate reader reading at a wavelength of 518 nm with a volume of 200 uL of each sample. The process was then repeated with an unmodified column.

### 2.5. Sample Preparation and Alkaline Hydrolysis

Standard stock solutions were prepared by dissolving corresponding amounts of fatty acids in chloroform (0.5 μmol/mL) and LPS in pyrogen-free water (2 mg/mL), separately. Sonication (15 min) was used to aid dissolution of LPS. Fatty acids, either 3-hydroxylauric acid, 3-hydroxymyristic acid, tridecanoic acid, pentadecanoic acid or heptadecanoic acid, and LPS standard solutions were then prepared by diluting with an appropriate amount of corresponding solvent. Upon alkaline hydrolysis, 250 μL of 16 M NaOH was added to a test tube containing 250 μL LPS solution (reaction solution of 8 M NaOH). The test tube was then capped and heated at 90 °C for 6 h. The solution was allowed to cool down to room temperature, after which pyrogen-free water (1 mL) was added. This solution was acidified with 12 M HCl with its acidity confirmed by pH paper. After that, the solution was extracted with ethyl acetate (2 × 3 mL). The combined organic layers were dried with sodium sulfate and transferred to a new test tube where the volatiles were removed under heating at 30 °C and a stream of air.

### 2.6. Fatty Acid Chain Derivatization

In a test tube, standard fatty acid solution was dried over low heat (37 °C) and a stream of air until the volatiles were removed and the fatty acids were recrystallized. Two different combinations of derivatizing agent were used for the derivatization of standard fatty acids and LPS dried extracts. Either derivatizing set 1 (50 µL MBTFA, 4 µL MTBSTA, and 10 µL pyridine) or set 2 (50 µL MSTFA and 10 µL pyridine) was added to each sterile test tube containing different quantities of dried contents. Since MTBSTA and MSTFA undergo hydrolysis with moisture easily, syringes were used to withdraw the agents directly from the storage bottle through the PTFE/silicone septum without opening the cap. The test tubes were then screw-capped and heated at 65 °C for 1 h. After cooling down to room temperature, solutions were collected for GC-MS analysis.

To optimize the use of the internal standard, 3-hydroxymyristic acid was prepared in a series of 8 different concentrations between 2 × 10^−7^ and 5 × 10^−6^ M. To each test tube containing 3-hydroxymyristic acid, 60 µL of 1 × 10^−6^ M tridecanoic acid, pentadecanoic acid or heptadecanoic acid was added. The mixture was then dried over low heat (37 °C) and a stream of air. The derivatization of dried content was performed as mentioned above using 50 µL MSTFA and 10 µL pyridine.

The temperature profile for the GC-MS separation of fatty acid derivatives with split/splitless injection is as follows: The injector temperature was 280 °C. The oven temperature was initially held at 100 °C for 1 min, and then ramped at a rate of 15 °C/min until reaching a final temperature of 280 °C. The oven was then held at 280 °C for 2 min, resulting in an overall separation time of 15 min for a sample volume of 1 μL. Total ion chromatograms were also recorded to obtain mass spectra of the acid derivatives. GC-MS analysis was then performed in the selected ion monitoring (SIM) mode for quantitative analysis with combinations of *m*/*z* = 142, 233, 255, 271, 283, 285, 299, 341, 345, and 373, which were found to be fragments of the fatty acids.

### 2.7. Determination of LPS-PMB Interaction in Blood

Glass beads with an average surface area of 5.2 × 10*^−^*^4^ cm^2^ were prepared, modified, and characterized according to previously published methods and results [39]. LPS-spiked whole blood (800 ng/mL or 1 ng/mL, 2 mL) was added to PMB-modified glass beads (250, 500 or 1000 units) in a sterile vial. These concentrations were chosen to be at the extreme ends of high and low concentration to compare LPS interactions with the beads to the existing fibers. The vial was capped, wrapped with aluminum foil and vortexed (220 rpm) at 37 °C for 90 min. The same protocol was followed for another four controls: incubation of LPS-unspiked donor blood alone or in presence of PMB-modified beads and incubation of LPS-spiked blood alone or in presence of unmodified glass beads. After incubation, the blood samples were transferred to sterile polypropylene tubes and centrifuged at 2500 rpm for 10 min, allowing the separation of plasma from blood cells. The collected plasma aliquots (500 μL) were then mixed with fresh naïve blood (5 mL). This diluted fluid was incubated for an additional 90 min at 37 °C under vortexing at 220 rpm. A final centrifugation at 2500 rpm for 10 min was performed, then plasma extract was collected for determination of TNFα levels using a 96-well human TNFα ELISA kit purchased from Thermo Scientific (Waltham, MA, USA).

The plasma samples were added to an anti-human TNF-α precoated strip plate followed by incubation with biotinylated antibody reagent, streptavidin-horseradish peroxidase (HRP) reagent and TMB substrate individually. ELISA plates were read at a wavelength of 450 nm with reference at 630 nm using an automatic ELISA photometer. A corresponding calibration curve of TNFα (0–500 pg/mL) was constructed each time the ELISA kit was used. The remaining PMB-modified beads were rinsed with steric saline (10 × 5 mL ). 1 mL pyrogen-free water was added to the beads and underwent alkaline hydrolysis (with the use of 1 mL 16 M NaOH instead) and derivatization with procedures as presented above. GC-MS analysis of derivatized fatty acid chains of LPS allows further determination of the amount of LPS being bound to PMB-modified beads from the calibration curve constructed with a wide range of LPS and fatty acid standards.

## 3. Results and Discussion

### 3.1. Fluorescence Study of Interaction of LPS with Cartridge Material

A 0.7 g sample of PMB-modified fiber was incubated with 0.2 mg of fluorescent-labelled LPS (FITC-LPS). This large amount of LPS was used to ensure incomplete binding, allowing a comparison to be made between the fibers and later the glass beads. After incubation, an increase in fluorescent response was observed in the extracted GdmCl solution. The corresponding signal was equivalent to 8 μg/mL of FITC-LPS, or a total of 40 μg of LPS, suggesting that the PMB-modified fiber was able to bind 2% of the presented FITC-LPS. The low value is likely associated with the small size of the sample compared to the concentration of LPS employed. Nevertheless, this result confirms some binding of the toxin by the fiber occurred.

The next step in examining the effectiveness of the cartridge was a study of the potential role played by non-specific adsorption to the base polystyrene material. Proteinase K, a serine protease, was used to cleave peptide bonds in PMB in order to eliminate its ability to capture or neutralize LPS, consequently enabling the measurement of the occurrence of non-specific adsorption of LPS to the column matrix. Simulation of extracorporeal LPS removal in whole blood via the “baby” cartridge was performed. In a sample of 170 μg of FITC-LPS, a completely unmodified column bound 10.0 μg of FITC-LPS, while the protease K-treated column bound 3.8 μg of FITC-LPS. Clearly, a significant amount of FITC-LPS was observed to bind to the proteinase K-treated column (38% of the unmodified column result). This suggests that the non-specific adsorption of LPS does play a role in the removal of LPS from the bloodstream.

### 3.2. Glass Beads and LPS Bioactivity Based on Human Tumor Necrosis Factor α (TNF-α) Production

The successful formation of mixed-SAMs and attachment of PMB molecules to the glass bead surface were confirmed from angle-resolved XPS results. After PMB immobilization, characteristic peaks corresponding to OEG-TTTA and MEG-TFA (fluorine) nearly disappear and characteristic peaks corresponding to the PMB probe (nitrogen) rise, as described in prior research [39]. The average radius of each bead is 0.05 cm with a corresponding surface area of 5.2 × 10^−4^ cm^2^. Blood samples being used throughout the study were collected on the same day of each experiment, without delay.

Pro-inflammatory mediators such as tumor necrosis factor (TNF), interleukin (IL)-1, IL-6, and IL-8 are released from blood cells as a result of the introduction of LPS to the bloodstream. By monitoring the change in TNF-α level in blood samples before and after the addition of LPS, it is possible to evaluate the bioactivity of LPS. Significantly elevated production of TNF-α in blood suggests that LPS is present as a highly bioactive and therefore toxic form (aggregates).

A total of four control experiments were used in order to investigate the role PMB-modified glass beads might play in reducing LPS in blood. The first two controls were the deposition of whole blood and LPS-spiked blood in two separate empty sterile vials. The former served as a blank, while the latter provided a baseline for the effects of LPS on the production of TNF-α during experiments. The incubation of LPS-spiked blood in unmodified glass beads served as a third control to study whether the non-specific adsorption of LPS might contribute to TNF-α production. The last experiment was the incubation of unmodified whole blood with PMB-modified glass beads. These experiments were intended to confirm that LPS was responsible for any observed elevations in the production of TNF-α.

**Figure 5:**
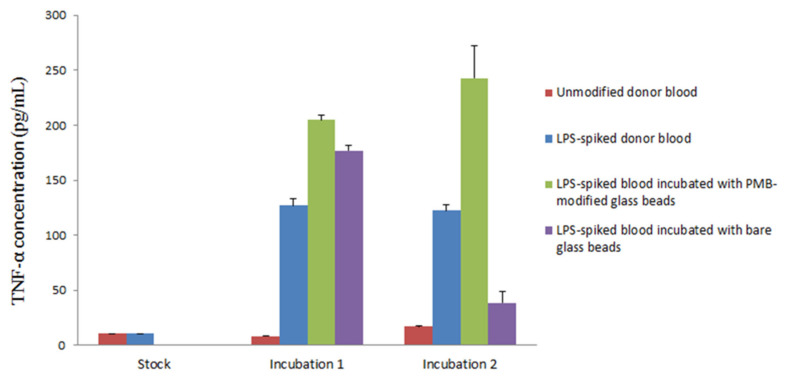
Production of TNF-α cytokine upon two incubations. 1: Incubation of LPS-spiked donor blood with PMB-modified glass beads (green) and bare glass beads (purple). 2: Incubation of plasma samples collected from incubation 1 with naïve blood. Additional unmodified donor blood (red) and LPS-spiked donor blood (blue) were incubated individually as sample controls. (Triplicate analysis. One significant reading for each sample is shown along with standard deviation).

The incubation of LPS-spiked blood samples was completed in two steps. First, the blood was incubated in a sterile vial with a batch of 1000 PMB-modified glass beads. The blood was then separated from the beads and centrifuged to collect a plasma layer for a second incubation in naïve blood. Naïve blood was used as a source of white blood cells to stimulate TNF-α production. Second, the plasma samples were added in triplicate to an anti-human TNF-α precoated ELISA strip plate. The samples were subsequently incubated with biotinylated anti-body reagent, streptavidin-horseradish peroxidase (HRP) reagent and TMB substrate individually for immunoassay conduction. The interaction between HRP and the TMB substrate yielded a blue solution that turned yellow when Stop Solution (sulfuric acid) was added. A standard curve of TNF-α (0–500 pg/mL) was constructed each time the ELISA strip plate was used for the quantification of TNF-α in samples (Figure S1), yielding the following data:

Absorbance = (363 ± 8) × 10^−5^[TNF-α] + (0.07 ± 0.01), R^2^ = 0.9941, where [TNF-α] is the concentration of TNF-α in pg/mL. Using the established TNF-α standard curve, it is possible to calculate the amount of TNF-α produced in each sample during different points in the experiment. Figure 5 shows the result from one of the triplicates tested in the experiment along with the associated standard derivation. Plasma samples collected from unmodified whole donor blood, which served as blank control, had a TNF-α concentration of 14.1 pg/mL, suggesting a negligible presence of TNF-α and low LPS activity as expected. Samples collected from LPS-spiked blood immediately after LPS was mixed with whole blood exhibited a TNF-α concentration of 13.9 pg/mL. This low concentration, which is comparable to readings taken from samples of unmodified whole blood, can be explained by the incomplete activation of TNF-α. Incubation of unmodified and LPS-spiked whole blood at 37 °C for 90 min resulted in TNF-α concentrations of 8.3 and 126.9 pg/mL, respectively. While unmodified blood samples still showed insignificant production of TNF-α, a more than tenfold increase was observed in LPS-spiked blood samples. This shows that the bioactive form of LPS was present in the sample with the resultant triggering of TNF-α production during the incubation.

Notably, stimulated production of TNF-α was observed when LPS-spiked blood was incubated with bare glass beads or PMB-modified glass beads. Both samples contained a higher concentration of TNF-α compared to the LPS-spiked blood control. These results might be due to the extra surface area provided by the glass beads, which encouraged the interaction of LPS with blood and therefore stimulated higher TNF-α production.

PMB-modified glass beads are clearly capable of the minimization or termination of LPS-triggered TNF-α production. Based on this observation, there are two possible mechanisms at play. First, it is possible that LPS has been removed from the blood samples, as indicated in other studies. If the LPS was captured by the PMB beads, it would no longer activate the production of TNF-α, leading to a natural drop in TNF-α concentration. Second, alternatively, it is possible that the PMB-modified glass beads are capable of producing a neutralization mechanism in which PMB triggers the monomerization of LPS bioactive aggregates into a non-toxic form, thereby preventing the production of TNF-α.

In order to clarify the role that PMB-modified glass beads play in reducing TNF-α production, it is obviously necessary to examine whether the surfaces of these beads actually capture LPS. Therefore, an assay involving alkaline hydrolysis and derivatization was performed with PMB-modified glass beads collected after incubation with LPS-spiked blood. Before describing the results of this work, the newly developed analytical technique for this assay is described in detail.

### 3.3. LPS Assay Based on Derivatized Released Fatty Acids from LPS

As indicated in Figure 3, the two fatty acids, 3-hydroxylauric acid and 3-hydroxymyristic acid, were designated the likely species to be released from Lipid A of LPS by alkaline hydrolysis. Gas chromatography-mass spectrometry (GC-MS) was employed to analyze the MBTFA, MTBSTFA, and MSTFA derivatized products of the two fatty acids. Mass spectra of the derivatives of acids using MBTFA and MTBSTFA share a common characteristic: the elimination of trifluoroacetic acid along with the tert-butyl group and two methyl groups of the silyl functionality, resulting in a base peak for the M-171 fragment. Mass spectra of products collected from fatty acid derivatization using MSTFA exhibited a peak at M-15 and a base peak at M-155 or M-127, suggesting the complete elimination of the nonyl functional group followed by the loss of a methyl group.

GC separation of acid derivatives was then performed using the mass-to-charge ratio collected from the previous MS scan for mass selective detection with selected ion monitoring and split/splitless injection (Figure S2). MSTFA as a derivatizing agent yields a cleaner chromatogram with better resolution and was thus chosen to produce a calibration curve for 3-hydroxylauric acid and 3-hydroxymyristic acid derivatives (Figure S3). The results of linear fitting via Origin Pro 8 for quantification are shown in Table 1.

In order to further evaluate the analytical method for the fatty acids, the usefulness of internal standards, three saturated fatty acids (tridecanoic acid, pentadecanoic acid and heptadecanoic acid), were selected for study based on similar structures, derivatization and GC retention time as 3-hydroxylauric acid and 3-hydroxymyristic acid. (An example with heptadecanoic acid is depicted in Figure S4). Linear fits were obtained which could be employed for quantification:

- Heptadecanoic acid: y = 0.9441x − 0.1482, R^2^ =0.997
- Pentadecanoic acid: y = 0.1167x + 0.0189, R^2^ =0.948
- Tridecanoic acid: y = 0.3111x − 0.8124, R^2^ = 0.877

In these equations y is the ratio of peak area of 3-hydroxymyristic acid derivatives to that of the internal standard, and x is the ratio of concentration of 3-hydroxymyristic acid to that of the internal standard.

Following the successful development of a protocol for fatty acid analysis, a practical assay of LPS from *E. coli* serotype O55:B5 was performed. This was achieved by alkaline hydrolysis, which involves the cleavage of the ester or amide bond, which connects the 3-hydroxy fatty acids to the lipid A moiety.

This procedure was selected instead of acidic hydrolysis because it has been shown that this form of hydrolysis minimizes side reactions [44]. The hydrolysis of LPS was performed using 8M NaOH incubation for 6 h. The prolongation of hydrolysis time (up to 10 h) did not produce a statistically significant increase in the yield of free 3-hydroxy fatty acids. Derivatization was then performed via an analogous procedure to that described above. Again, the resulting products were analyzed by GC-MS. A calibration curve for LPS derivatives (Figure S5) with LOD = 0.04 µg/mL and LOQ = 0.13 µg/mL was also conducted using the GC-MS results:

Peak area = (2.79 ± 0.04) × 10^5^ [LPS] – (3.6 ± 0.3) × 10^4^_,_ R^2^ = 0.9945

where [LPS] is the concentration of LPS in µg/mL.

The mass spectrum of the main peak of the gas chromatogram for LPS derivatives reveals that 3-hydroxymystric acid is the main product of the hydrolysis process. For each LPS molecule, two molecules of 3-hydroxymystric acid are generated from the cleavage of corresponding ester bonds of the lipid A moiety.

### 3.4. Free and Bound LPS on Interaction with PMB-Modified Glass Beads

The alkaline hydrolysis and derivatization protocol for the GC-MS assay (for 3-hydroxymyristic acid derivatives) as described above was conducted on both bare and PMB-modified glass beads separated and collected after incubation with LPS-spiked blood (2 mL of 0.8 μg/mL LPS-spiked blood was used in each treatment). To prevent contamination arising from remaining blood, the beads were carefully rinsed with sterile saline. The bare glass bead experiment was performed to examine the possibility of non-specific adsorption of LPS on the bare bead surface. The measurements shown in Table 2 reveal that PMB-modified glass beads only captured 1.43 ± 0.05% of LPS presented in the blood samples, while the bare glass beads captured 0.78 ± 0.05%. These measurements support two key findings. First, the bare glass beads were able to capture approximately half the amount of LPS compared to the PMB-modified glass beads, suggesting that the LPS-PMB interaction does not constitute a significant process. Second, the bare glass beads did capture some LPS, indicating that the non-specific adsorption of LPS on their surface did play a minor role in the removal of LPS from blood samples.

**Figure 6:**
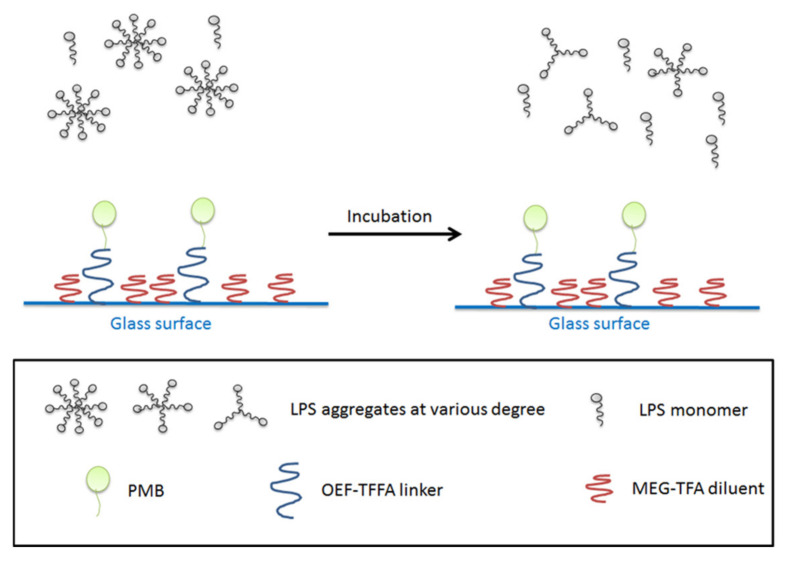
LPS aggregates before incubation with PMB-modified glass beads (**left**), after incubation (**right**). The majority of LPS exists in form of monomers or aggregates with less LPS molecules per unit. The bottom panel shows the legends being used in the figure.

The binding interaction between PMB-modified glass beads and LPS was clearly limited according to the calculated amount of captured LPS. It is also possible that the LPS-PMB bonds were so weak that the act of rinsing the glass beads with saline in fact broke the bonds. This result implies that instead of a physical LPS-PMB bond, the interaction between the two entities is more likely to be a long-distance interaction at several sites of the molecules. Accordingly, we suggest that the disaggregation of LPS molecules as a result of such interaction actually decreases the bioactivity of LPS, which was observed earlier from a decrease in TNF-α production (Figure 6). This mechanism stands in contrast to indications in the literature that outright LPS capture occurs [45]. Both theoretical and experimental research has confirmed that the disintegration of micelles and vesicles is possible at the solid-liquid interface and that there is a dependence on the free energy properties of the solid surface under study [46,47,48]. It would clearly be anticipated, in this case, that the presence of PMB would enhance this process. Moreover, the antifouling surface design is likely to significantly reduce any surface adsorption of the monomer.

**Figure 7:**
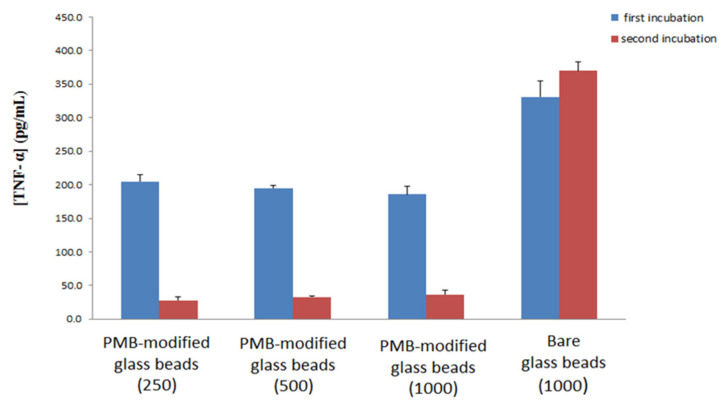
Production of TNF- α cytokine upon two incubations: First incubation (blue) of LPS-spiked donor blood with PMB-modified glass beads or bare glass beads and subsequent incubation with naïve blood (red) with standard deviation. Numbers in brackets indicate number of glass beads used for the first incubation.

The experiment was repeated for different amounts of PMB-modified glass beads as well as for 1000 bare glass beads. Each set of beads was exposed to 1000 pg/mL of LPS-spiked blood Figure 7. The results of this work show that variation of the amount of PMB-modified glass beads does not significantly affect TNF-α production, again supporting the hypothesis that PMB acts to interrupt LPS micelles as opposed to directly removing LPS from the blood. Previous research has shown that LPS forms large aggregates normally, and falls into smaller more lamellar forms following interaction with PMB [49]. The work presented here would suggest that these interactions are the driving force of PMB based cartridges for treating sepsis, as opposed to direct removal of LPS from the blood, however further work would be needed to determine if this is the case. In addition, the ability of PMB-modified glass beads to reduce the level of LPS in blood compared to the case of non-modified bare glass beads is confirmed, as the bare glass beads showed no reduction in activity after incubation in naïve blood.

### 3.5. Comparison of Fiber Column with PMB-Modified Glass Bead System for Potential Endotoxin Treatment

The direct comparison of PMB-immobilized fiber columns to PMB-modified glass beads in terms of their relative LPS binding capacity is not apparent in the present work. It is at least possible, however, to compare the LPS neutralization capability of a PMB-immobilized fiber column to that of PMB-modified glass beads by calculating the number of beads required to achieve the same outcome via cartridge hemoperfusion.

Current findings suggest that the complete neutralization of LPS from the bloodstream (~4340 mL) of a patient displaying a high LPS level of 1000 pg/mL would require approximately 1.89 × 10^5^ PMB-modified glass beads [50]. Such a quantity of beads would occupy a volume of 98.8 cm^3^ if packed in a flow-through hemoperfusion cartridge similar to the Toraymyxin cartridge. A regular adult fiber cartridge has a length of 225 mm and diameter of 49 mm. In comparison, a corresponding quantity of PMB-modified beads in a packed cylinder with the same diameter would only require a length of 5.2 mm [51].

## 4. Conclusions

This work examines the feasibility of substituting PMB-modified glass beads for the commercially available PMB-immobilized polystyrene-based fiber system that is currently in use in cartridges for the reduction of LPS in blood. PMB-modified glass beads were shown to be able to reduce LPS in blood as efficiently as the PMB-immobilized fiber column, using a much smaller volume of beads compared to fiber. This reduction would allow cartridges that are 43 times shorter to be used, allowing for a reduction in materials and lower production costs for these cartridges. The analysis conducted here also suggests that PMB does not physically capture or significantly remove LPS from the blood samples, and may act to disrupt LPS micelles and reducing their toxicity in that way. However further work is needed to determine if this is the case, including measuring the amount of LPS micelles present before and after interaction with PMB, as well as testing PMB itself in a non-surface bound state.

## Figures and Tables

**Table 1:** Linear fit for calibration of 3-hydroxylauric acid and 3-hydroxymyristic acid derivatives formed with MSTFA.

.	n	Slope (sd)	Intercept (sd)	R^2^	LOD (M)	LOQ (M)
3-hydroxylauric acid	9	7.7(4) × 10^11^	−4.2(9) × 10^6^	0.990	2.5 × 10^−8^	5.5 × 10^−7^
3-hydroxymyristic acid	14	1.14(2) × 10^12^	−1.2(4) × 10^6^	0.994	7.5 × 10^−9^	1.0 × 10^−7^

Note: n: number of samples; sd: standard derivation; R^2^: coefficient of determination; LOD: limit of detection; LOQ: limit of quantification; M: molar concentration.

**Table 2:** Capture of LPS by bare and PMB-modified glass beads *.

Bead (Incubated with 2.0 μg/mL LPS in Blood)	Peak Area	[LPS] (ng/mL)	Captured Amount of LPS (ng)
Bare glass beads	21,097	206 ± 10	12.4 ± 0.7
PMB-modified glass beads	68,497	383 ± 10	22.9 ± 0.8

* Peak area from chromatogram.

## Data Availability

Data is contained within the article or Supplementary Materials.

## Supplementary Materials

The following are available online at https://www.mdpi.com/article/10.3390/ma15041551/s1, Figure S1: Calibration standard curve for human TNF-α analysis, Figure S2: Overlapped gas chromatograms of 5 × 10^−6^ M 3-hydroxy fatty acid derivatives using MSBFA and MTBSTFA (blue), or MSTFA (blank) as derivatizing agents (single ion monitoring), Figure S3: Calibration curves for 3-hydroxylauric acid and 3-hydroxymyristic acid derivatives, Figure S4: Calibration curve for 3-hydroxymyristic acid using heptadecanoic acid as internal standard, Figure S5: Calibration curve for derivatized fatty acids from LPS.
