# Supplementary material for: Interaction of Lipopolysaccharide-Spiked Blood with Anti-Fouling Polymyxin B-Modified Glass
Source: Materials (Basel). 2022 Feb 18;15(4):1551. doi: 10.3390/ma15041551 (PMC8876862; doi:10.3390/ma15041551)
Supplement: Supplementary file 1 [file materials-15-01551-s001.zip › materials-1517285-supplementary.pdf]

materials

MDPI

Supplementary Materials

Interaction of Lipopolysaccharide-Spiked Blood with Anti-Fouling Polymyxin B-Modified Glass

Hoi Ting Wong [1], Alexander Romaschin [2], Sara Bjelobrk [1], Brian De La Franier [1] and Michael Thompson [1,*]

[1] Department of Chemistry, University of Toronto, 80 St. George Street, Toronto, ON M5S 3H6, Canada; hoiting.wong@mail.utoronto.ca (H.T.W.); sara.bjelobrk@mail.utoronto.ca (S.B.); brian.delafranier@mail.utoronto.ca (B.D.L.F.)

[2] Clinical Biochemistry, St. Michael's Hospital, 30 Bond Street, Toronto, ON M5B 1W8, Canada; romaschina@gmail.com

* Correspondence: m.thompson@utoronto.ca; Tel.: +1-416-978-3575

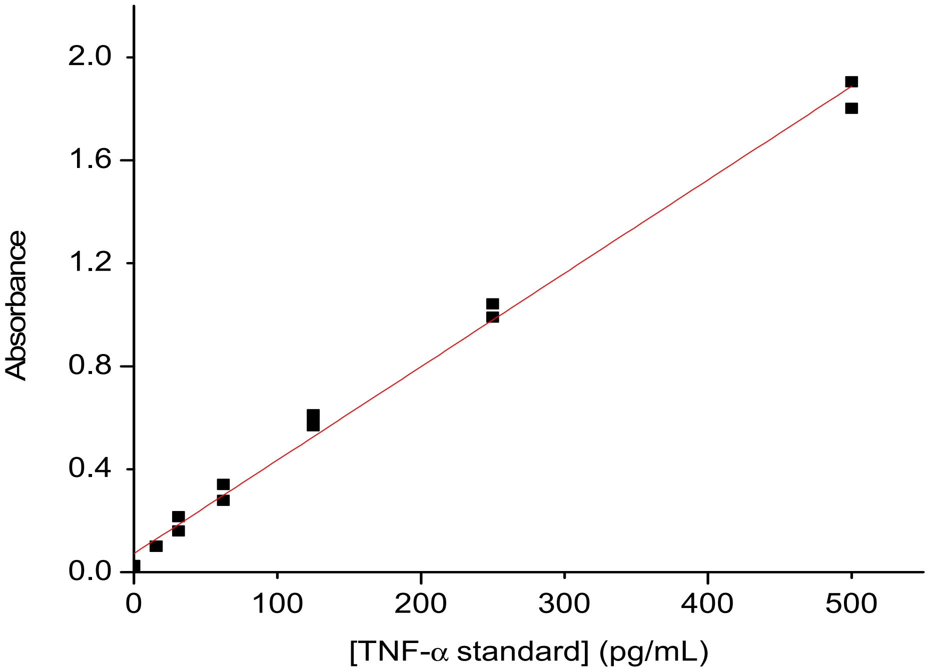

Figure S1. Calibration standard curve for human TNF-α analysis.

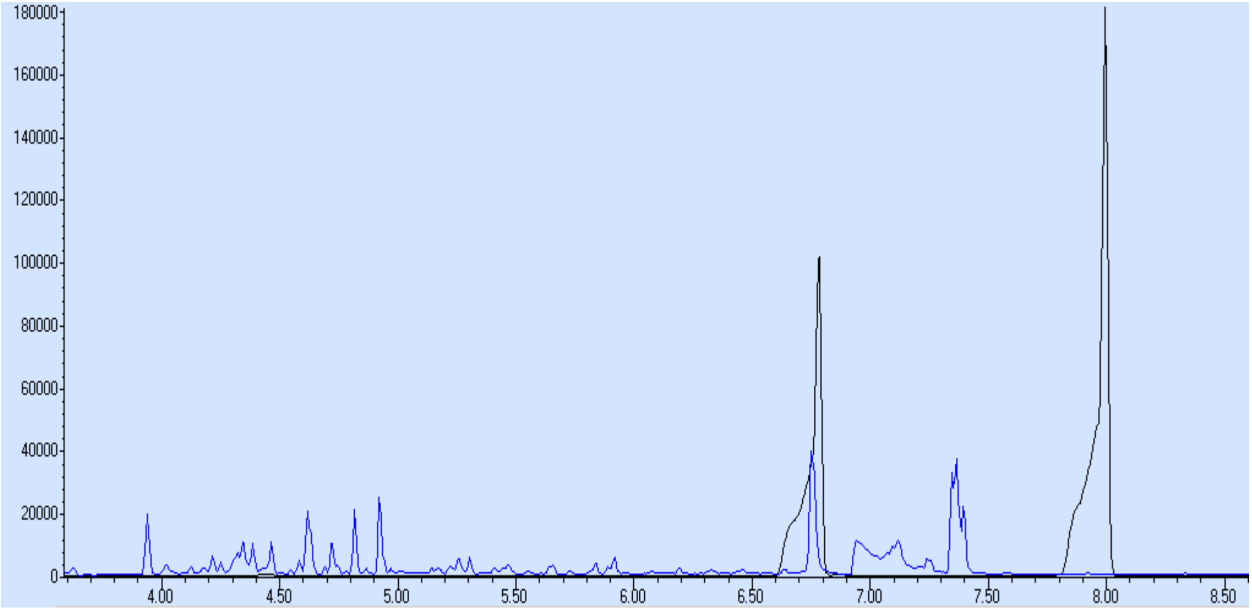

Figure S2. Overlapped gas chromatograms of 5 × 10^{-6} M 3-hydroxy fatty acid derivatives using MSBFA and MTBSTFA (blue), or MSTFA (blank) as derivatizing agents (single ion monitoring).

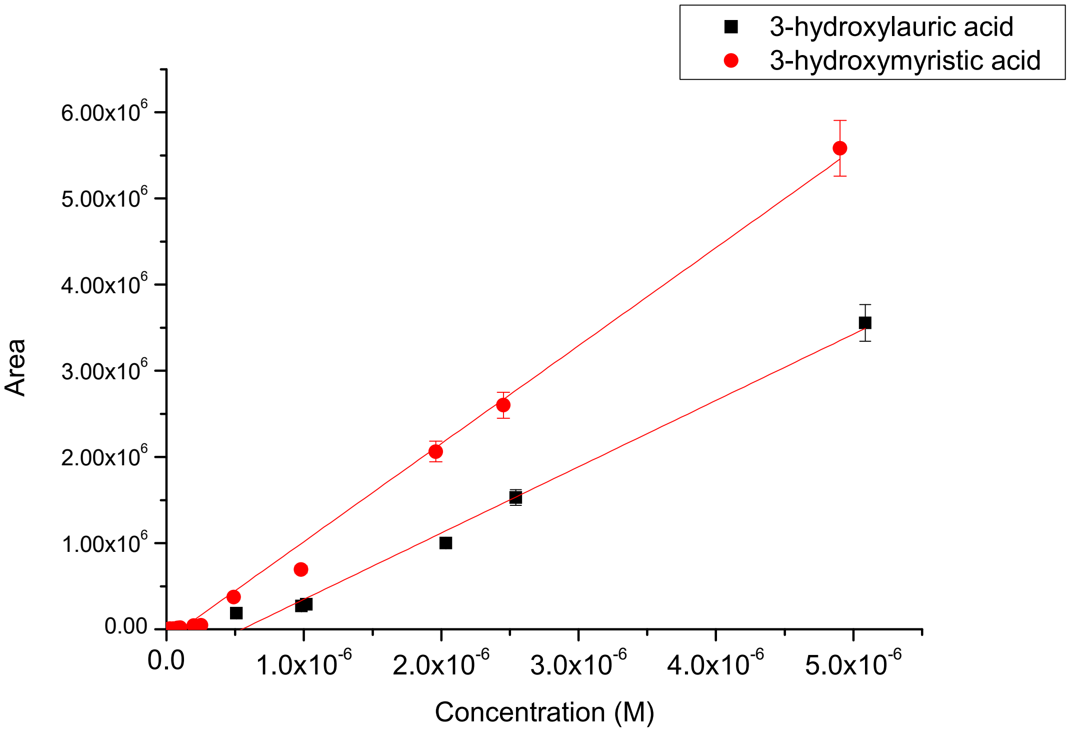

Figure S3. Calibration curves for 3-hydroxylauric acid and 3-hydroxymyristic acid derivatives.

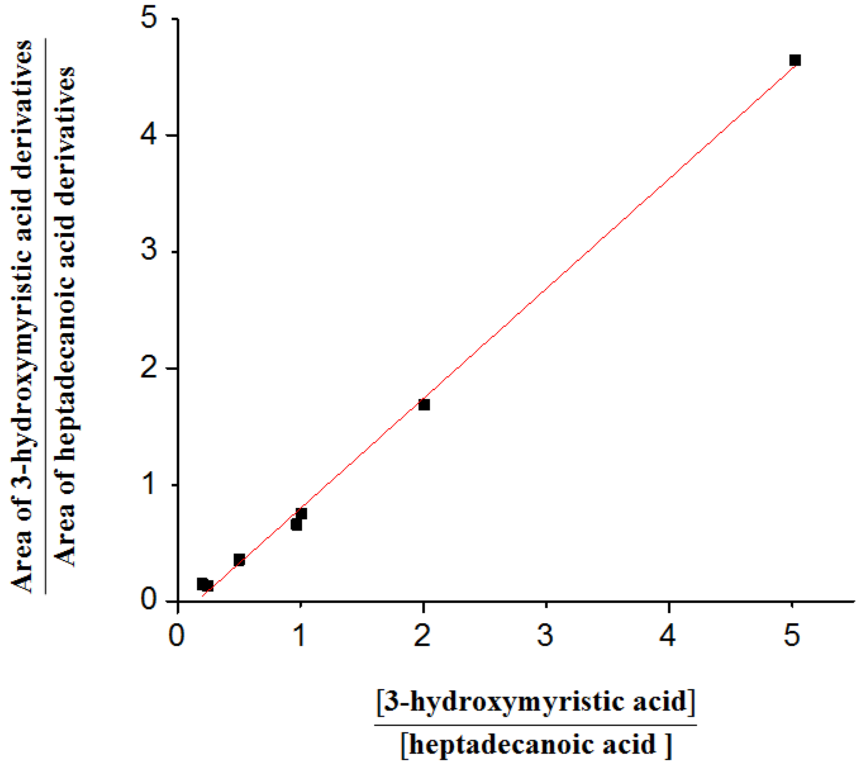

Figure S4. Calibration curve for 3-hydroxymyristic acid using heptadecanoic acid as internal standard.

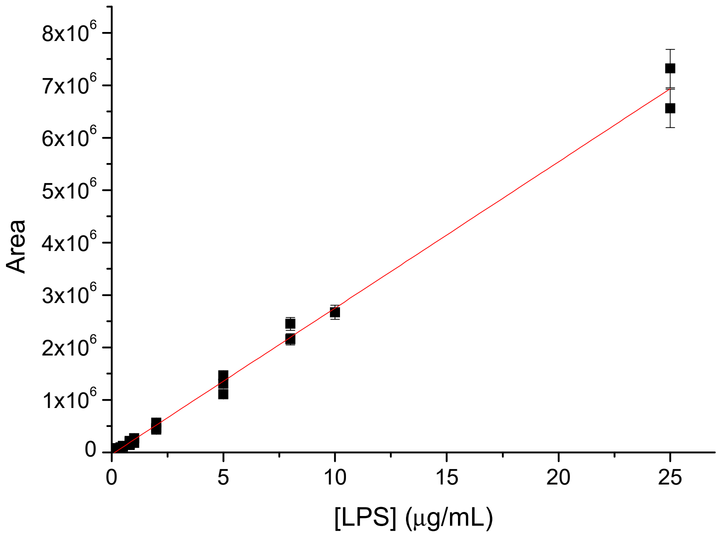

Figure S5. Calibration curve for derivatized fatty acids from LPS.
